# Calcium imaging with genetically encoded sensor Case12: Facile analysis of α7/α9 nAChR mutants

**DOI:** 10.1371/journal.pone.0181936

**Published:** 2017-08-10

**Authors:** Irina Shelukhina, Ekaterina Spirova, Denis Kudryavtsev, Lucy Ojomoko, Markus Werner, Christoph Methfessel, Michael Hollmann, Victor Tsetlin

**Affiliations:** 1 Department of Molecular Basis of Neurosignalling, Shemyakin-Ovchinnikov Institute of Bioorganic Chemistry, Russian Academy of Sciences, Moscow, Russia; 2 Department of Biochemistry I, Ruhr University Bochum, Bochum, Germany; Weizmann Institute of Science, ISRAEL

## Abstract

Elucidation of the structural basis of pharmacological differences for highly homologous α7 and α9 nicotinic acetylcholine receptors (nAChRs) may shed light on their involvement in different physiological functions and diseases. Combination of site-directed mutagenesis and electrophysiology is a powerful tool to pinpoint the key amino-acid residues in the receptor ligand-binding site, but for α7 and α9 nAChRs it is complicated by their poor expression and fast desensitization. Here, we probed the ligand-binding properties of α7/α9 nAChR mutants by a proposed simple and fast calcium imaging method. The method is based on transient co-expression of α7/α9 nAChR mutants in neuroblastoma cells together with Ric-3 or NACHO chaperones and Case12 fluorescent calcium ion sensor followed by analysis of their pharmacology using a fluorescence microscope or a fluorometric imaging plate reader (FLIPR) with a GFP filter set. The results obtained were confirmed by electrophysiology and by calcium imaging with the conventional calcium indicator Fluo-4. The affinities for acetylcholine and epibatidine were determined for human and rat α7 nAChRs, and for their mutants with homologous residues of α9 nAChR incorporated at positions 117–119, 184, 185, 187, and 189, which are anticipated to be involved in ligand binding. The strongest decrease in the affinity was observed for mutations at positions 187 and 119. The L119D mutation of α7 nAChR, showing a larger effect for epibatidine than for acetylcholine, may implicate this position in pharmacological differences between α7 and α9 nAChRs.

## Introduction

Homopentameric α7 nicotinic acetylcholine receptors (α7 nAChRs) are ligand-gated ion channels (LGIC) characterized by a high calcium ion permeability [1] and a very fast desensitization rate [2]. Being present on both neuronal and non-neuronal cells, α7 nAChRs modulate different cellular processes, such as release of neurotransmitters, cytokines and neurotrophic factors, as well as downstream signaling, gene expression etc. [1, 3–5]. Expression of less abundant heteropentameric α7β2 nAChR was shown in basal forebrain [6]. Malfunctioning of α7 receptors is associated with neurodegenerative and psychiatric diseases, chronic pain, sepsis, rheumatoid arthritis etc. [7–9] That is why α7 nAChRs are attracting a strong interest as a target for drug discovery and design [7, 10–13], making studies of their molecular structure and functioning especially pertinent [14, 15].

α9 nAChR is close to α7 receptor in a number of properties: it is homopentameric, highly permeable to calcium ions and has a high-affinity to such antagonists as methyllycaconitine and α-bungarotoxin [16, 17]. Pharmacologically similar heteropentamers (α9α10 nAChRs) are formed in combination with α10 subunit [18]. α9-containing nAChRs are crucial for cholinergic efferent innervation of cochlear hair cells in inner ear [19], as well as for intensity and duration of some forms of chronic pain, such as mechanical hyperalgesia [20]. α-Conotoxins potently blocking α9 and α9α10 nAChRs are being developed as promising analgesics [12, 21, 22]. α9 receptor is a rather distant member of nAChR family and displays not only pharmacological properties of other Cys-loop receptors (GABA-A, glycine and 5HT3 receptors), but is also sensitive to muscarinic AChR ligands [17, 23]. Although acetylcholine activates α9-containing nAChRs, other classical nicotinic agonists such as nicotine, cytisine and epibatidine block them [17].

We wondered if certain amino acid residues which appear to be situated in the ligand-binding sites (judging from X-ray structures of complexes of acetylcholine-binding proteins, their chimeras and nAChR ligand-binding domains [24–26]) and which differ between α7 and α9 nAChRs, might be responsible for the unique pharmacology of the latter. To get an answer, we produced a series of α7 nAChR mutants with single amino acid substitutions in the orthosteric ligand-binding site.

Site-directed mutagenesis of nAChRs in combination with the two-electrode voltage clamp in *Xenopus* oocytes or patch clamp in mammalian cells is the gold standard for the most accurate determination of mutant pharmacology. However, α7 and α9 nAChRs are problematic for electrophysiology due to their difficult heterologous expression and fast desensitization [27, 28]. The problem is partly solved by employing easily-expressed and slowly-desensitizing chimeric receptors, which contain α7 or α9 nAChR extracellular domain and transmembrane domains of 5HT3- or glycine-receptors, and in general retain ligand affinities of the corresponding full-length nAChR [29–32]. Other helpful tools are “type II” positive allosteric modulators (PAM), which increase the probability of transient α7 nAChR activation by agonists, and also destabilize a ligand-bound nonconducting “desensitized” state of the receptor [33–35]. They are widely used in exquisite electrophysiological experiments and routine calcium imaging to amplify agonist-induced α7 nAChRs responses to the detectable level [36–39].

For efficient testing of α7/α9 nAChR mutant pharmacology, we have developed a calcium imaging technique based on the transient co-expression of α7 nAChR mutants, a chaperone (Ric-3 or NACHO), and the genetically-encoded calcium sensor Case12. It allowed us to explore the response of α7/α9 nAChR mutants to acetylcholine and epibatidine in the presence of PAM (PNU120596). The data obtained in this way correlated well with electrophysiological recordings, while the calcium imaging analysis was much simpler and faster.

## Materials and methods

### Molecular dynamics

To perform molecular dynamics study of the epibatidine-binding site for α7 nAChR and its mutants we used published the X-ray structure of the α7/AChBP chimera complex with epibatidine (PDB 3SQ6), using two adjacent subunits. The chosen α7/AChBP chimera residues were mutated in UCSF Chimera software. Forcefield parameters for the epibatidine molecule were generated *via* Swissparam tool. Models were energy minimized, equilibrated (100 ps of heavy atoms position restraint NVT equilibration, 100 ps of NPT equilibration) and simulated for 10 ns by unconstrained molecular dynamics with standard GROMACS 5.0 tools. The following parameters were used: Charmm 27 forcefield, short-range 1.0 nm electrostatic and van der Waals cutoffs, particle mesh Ewald long-range electrostatics and dodecahedron periodic cell with 1.2 nm of a protein-box distance.

### Site-directed mutagenesis

Mutagenic primers (Table 1) were designed using an online-program QuikChangePrimerDesign (Agilent Technologies, USA) with further manual optimization according to a previously published study of Zheng et al. [40]. PCR reactions were performed using Phusion Hot Start DNA polymerase (New England Biolabs, Canada) and an α7 nAChR-coding plasmid (human α7 nAChR-pCEP4, rat α7 nAChR-pcDNA 3.1/Hygro(+)) or α7/GlyR-pMT3 (chicken α7 nAChR extracellular domain fused with the human α1 GlyR transmembrane domain in pMT3 vector [32]) or mouse α1 nAChR-coding pRBG4 plasmid as a matrix DNA. PCR conditions were as follows: 98°C for 1 min, 25 cycles of 98°C for 10–30 s, 55–70°C for 1 min, 72°C for 3–4 min and extended at 72°C for 7 min. After performing the PCR reaction, the methylated parental DNA template was digested with the DpnI restriction enzyme (New England Biolabs, Canada) and XL1-Blue competent cells (Evrogen, Russia) were transformed with the PCR products. The desired nucleotide insertions were checked by sequencing of plasmids purified from 1–3 bacterial colonies.

**Table 1:** Mutagenic primers.

Primer	Nucleotide sequence 5’– 3’
**human α7 nAChR subunit**	
Q117T-F	5’-gcattgcacgtacctgcct-3’
Q117T-R	5’-aggcaggtacgtgcaatgc-3’
Y118W-F	5’-cattgccagtggctgcctcca-3’
Y118W-R	5’-tggaggcagccactggcaatg-3’
S184N-F	5’-gcaagaggattgaaaggttcta-3’
S184N-R	5’-tagaacctttcattcctcttgc-3’
E185V-F	5’-gcaagaggagtgtaaggttcta-3’
E185V-R	5’-tagaaccttacactcctcttgc-3’
F187S-F	5’-agtgaaaggtcttatgagtgc-3’
F187S-R	5’-gcactcataagacctttcact-3’
E189G-F	5’-aggttctatggatgctgcaa-3’
E189G-R	5’-ttgcagcattcatagaacct-3’
**rat α7 nAChR subunit**	
L119D-F	5’-cagtacgatcctccaggcatattc-3’
L119D-R	5’-ggaggatcgtactggcaatg-3’
**chimeric α7/GlyR subunit**	
E189A-F	5’-cttttatgcgtgctgtaaagaac-3’
E189A-R	5’-cttgactctcgaaaatacgcacgac-3’
**mouse α nAChR subunit**	
G153S-F	5’-ctatgacagctctgtggtggc-3’
G153S-R	5’-cacagagctgtcataggtccag-3’
Y190F-F	5’-gtgttcttctcctgctgccc-3’
Y190F-R	5’-gcaggagaagaacacccagtg-3’

### Neuroblastoma cell culture and transient transfection

Mouse neuroblastoma Neuro2a cells were purchased from the Russian collection of cell cultures (Institute of Cytology, Russian Academy of Sciences, Saint Petersburg, Russia). Cells were cultured in Dulbecco’s modified Eagle’s medium (DMEM, Paneco, Russia) supplemented with 10% fetal bovine serum (PAA Laboratories, Austria). They were sub-cultured the day before transfection and were plated at a density of 10000 cells per well (96-well plate) or 50000 cells per poly-D-lysine-coated glass coverslip (12 mm in diameter). On the next day Neuro2a cells were transiently transfected with plasmids coding α7 nAChR (human α7 nAChR-pCEP4, rat α7 nAChR-pcDNA 3.1/Hygro(+)) or its mutants, the chaperone Ric-3 (Ric3-pCMV6-XL5, OriGene, USA) or NACHO (TMEM35-pCMV6-XL5, OriGene, USA) and a fluorescent calcium sensor Case12 (pCase12-cyto vector, Evrogen, Russia) in molar ratio 4:1:1 following a lipofectamine transfection protocol (Invitrogen, USA). Wild-type or mutant mouse muscle α1β1δε nAChRs (pRBG4-vector) were expressed accordingly, not requiring a chaperone. The transfected cells were grown at 37°C in a CO_2_ incubator for 48–72 h, before binding and function were assessed.

### Fluorescent α-bungarotoxin binding assay

To assess cell expression of WT or mutant α7 and muscle nAChRs, the transfected Neuro2a cells plated on glass coverslips or on black 96-well plates with transparent glass or film bottom (Eppendorf, Germany) were stained with Alexa-Fluor 555-conjugated α-bungarotoxin (50 nM) for 20 min at room temperature. Cells were washed extensively with a buffer containing 140 mM NaCl, 2 mM CaCl_2_, 2.8 mM KCl, 4 mM MgCl_2_, 20 mM HEPES, 10 mM glucose; pH 7.4. to remove any unbound toxin. Fluorescent staining was observed with an epifluorescence microscope (Olympus, Japan). Controls were run simultaneously with 100-fold molar excess of unlabeled α-cobratoxin (purified from *Naja kaouthia* venom). Pictures were taken and processed with CellA Imaging Software (Olympus Soft Imaging Solutions GmbH, Germany) and open-source applications CellX and Image J.

### Cell viability assays

To determine Neuro2a cell viability after transfection with α7 nAChR, the chaperone NACHO and a calcium sensor Case12-coding plasmids, cells were incubated with 20 nM tetramethylrhodamine ethyl ester (TMRE, Invitrogen, USA) for 20 min and then washed with the buffer containing 140 mM NaCl, 2 mM CaCl_2_, 2.8 mM KCl, 4 mM MgCl_2_, 20 mM HEPES, 10 mM glucose; pH 7.4. Identification of the non-viable cells was performed by staining with propidium iodide (50 ng/ml, BD Biosciences, USA) for 5 min followed by brief washing with the buffer. The bright field and fluorescent pictures were taken with an epifluorescence microscope (Olympus, Japan) and processed with CellA Imaging Software (Olympus Soft Imaging Solutions GmbH, Germany) and open-source applications CellX and Image J.

### Single-cell Ca^2+^ imaging

For measurements of intracellular calcium concentration [Ca^2+^]_i_ changes, transfected Neuro2a cells plated on glass coverslips were perfused at room temperature with the buffer containing 140 mM NaCl, 2 mM CaCl_2_, 2.8 mM KCl, 4 mM MgCl_2_, 20 mM HEPES, 10 mM glucose; pH 7.4. Expression of Case12, a fluorescent genetically encoded sensor of calcium ions (ex/em = 491/516 nm), allowed direct monitoring of changes in [Ca^2+^]_i_ using an epifluorescence microscope with an appropriate filter combination and a CAM-XM10 cooled CCD camera (Olympus, Japan). Videos were made and processed using CellA Imaging Software (Olympus Soft Imaging Solutions GmbH, Germany), Image J, CellX, and OriginPro 7.5 software (OriginLab, MA, USA, for statistical analysis). The cells were exposed to acetylcholine iodide (Sigma, Germany), epibatidine (Tocris, UK), and α-cobratoxin purified from *Naja kaouthia* venom, and changes in Case12 fluorescence were recorded from each cell independently. To increase the registered changes, all ligand solutions contained the α7 nAChR positive allosteric modulator PNU120596 (10 μM, Tocris, UK). To allow recovery of the cells, the washing steps lasted 5–10 minutes. All recordings were made at room temperature.

### Ca^2+^ measurements for cell population

Transfected Neuro2a cells were grown on black 96-well plates (Corning, USA) at 37°C in a CO_2_ incubator for 72h, then growth medium was removed and cells were washed with buffer containing 140 mM NaCl, 2 mM CaCl_2_, 2.8 mM KCl, 4 mM MgCl_2_, 20 mM HEPES, 10 mM glucose; pH 7.4. Cells expressing nAChRs and the fluorescent calcium sensor Case12 were proceeded directly, but alternatively Neuro 2a cells expressing the receptor of interest were loaded with a fluorescent dye Fluo-4, AM (1.824 µM, ThermoFisher Scientific, USA) and water-soluble probenecid (1.25 mM, ThermoFisher Scientific, USA) for 30 min at 37°C and then were kept for 30 min at room temperature according to the manufacturer’s protocol.

Cells were incubated with the α7 nAChR positive allosteric modulator PNU120596 (10 μM, Tocris, UK) for 20 min at room temperature before acetylcholine (Sigma, Germany) or epibatidine (Tocris, UK) addition. To assess muscle nAChRs (WT or mutant), this step was skipped. The plates were transferred to the multimodal microplate reader Hidex Sence (Hidex, Turku, Finland) where the cells were excited by light of 485 nm wavelength and emitted fluorescence was detected at 535 ± 10 nm. Fluorescence was recorded every 2 s for three minutes following agonist addition. Responses were measured as peak intensity minus basal fluorescence level, and are expressed as a percentage of the maximal response obtained to agonist. Data files were analysed using Hidex Sence software (Hidex, Turku, Finland) and OriginPro 7.5 software (OriginLab, MA, USA, for statistical analysis). Controls were run in the presence of 4 μM α-cobratoxin.

### Whole-cell patch clamp

Transfected Neuro2a cells were immersed in recording buffer (20 mM HEPES, 140 mM NaCl, 2.8 mM KCl, 2 mM CaCl_2_, 1 mM MgCl_2_, 10 mM glucose; pH 7.4) and subjected to whole-cell patch clamp with the aid of HEKA amplifier (HEKA Elektronik, Germany). Micropipettes were pulled on a PC-10 instrument (Narishige, Japan) from borosilicate glass capillaries (Harvard Apparatus, USA) and filled with internal buffer (140 mM CsCl, 6 mM CaCl2, 2 mM MgCl2, 2 mM MgATP, 0.4 mM NaGTP, 10 mM HEPES/CsOH, 20 mM BAPTA/KOH; pH 7.3). During a typical experiment, acetylcholine at concentrations from 1 to 100 μM was applied to a cell through a Fast-Step perfusion system (1 ml/min, Warner Instruments, US) along with PNU120596 (Tocris, UK) given at 10 μM. Electrophysiological recordings were performed at a holding potential of -40 mV. Currents were recorded through Patchmaster software (HEKA Elektronik, Germany) and analyzed using OriginPro 7.5 (OriginLab, USA). Current amplitudes were normalized to a maximal response for each cell.

### Two-electrode voltage clamp

Rat α7 nAChR and α7 nAChR [L119D] in the pSGEM vector were linearized using XbaI (Promega, USA). mRNAs were transcribed *in vitro* using the T7 mMessage mMachine (Ambion Inc., Austin, TX, USA) transcription kit. RNAs were purified by phenol:chloroform extraction and isopropanol precipitation. Ovary tissue from adult female *Xenopus laevis* was cut into small pieces and these pieces were digested with collagenase A (4 mg mL^-1^, Worthington, USA) in Barth’s solution without calcium (88.0 mM NaCl, 1.1 mM KCl, 2.4 mM NaHCO_3_, 0.8 mM MgSO_4_, 15.0 mM HEPES/NaOH, pH 7.6) for 1.5–2 h at 20°C. The oocytes were stored in Barth’s solution with calcium (88.0 mM NaCl, 1.1 mM KCl, 2.4 mM NaHCO_3_, 0.3 mM Ca(NO_3_)_2_, 0.4 mM CaCl_2_, 0.8 mM MgSO_4_, 15.0 mM HEPES/NaOH, pH 7.6) supplemented with 63.0 μg mL^-1^ penicillin-G sodium salt, 40.0 μg mL^-1^ streptomycin sulfate, 40.0 μg mL^-1^ gentamicin. Stage V–VI oocytes were selected and injected with 5 ng mRNA of rat α7 nAChR or α7 nAChR [L119D] in a total injection volume of 15 nl. To obtain expression of α7/GlyR, the oocytes were placed in Ca^2+^-free ND96 electrophysiological buffer for 30 min at room temperature and then were kept at 18°C in the full ND96 buffer (96 mM NaCl, 2 mM KCl, 1.8 mM CaCl_2_, 2 mM MgCl_2_, 5 mM HEPES/NaOH, pH 7.6), and plasmids coding the WT α7/GlyR or mutant α7/GlyR [E189A] were injected into the nuclei of oocytes using an Auto-Nanoliter Injector NanoJect-2 (Drummond Scientific). About 3 ng of plasmid were injected into each oocyte nucleus in a total injection volume of 32 nl.

After injection, oocytes were incubated at 18°C in the full ND96 buffer or in Barth’s solution with calcium for 48–120 h. Electrophysiological recordings were made using a Turbo TEC-03X amplifier (Npi electronic, Germany) and WinWCP recording software (University of Strathclyde, UK). Oocytes were placed in a small recording chamber with a working volume of 50 μl, and 50–100 μl of agonist (acetylcholine, nicotine, or epibatidine) solution in ND96 electrophysiological buffer or Ba^2+^ Ringer’s solution (115.0 mM NaCl, 2.5 mM KCl, 1.8 mM BaCl_2_, 10.0 mM HEPES/NaOH, pH 7.2) were applied to an oocyte. To allow receptor recovery from desensitization, the oocytes were superfused for 5–10 min with buffer (1 ml min^-1^) between ligand applications. Electrophysiological recordings were performed at a holding potential of -60 mV.

### Data and statistical analysis

The data and statistical analysis in this study comply with the recommendations on experimental design and analysis in pharmacology [41]. Data are reported as mean ± SD, mean (95% confidence interval) or mean ± SEM for the indicated number of independent experiments and processed cells. Statistical analysis (two-tailed Student’s *t*-test,one-way ANOVA, Shapiro-Wilk test of normality of distribution, and Pearson correlation) was performed using OriginPro 7.5 (OriginLab Corporation, Northampton, MA, USA). In all the tests, *p* < 0.05 was taken as significant.

## Results

### Site-directed mutagenesis of the α7 nAChR ligand-binding site

**Fig 1:**
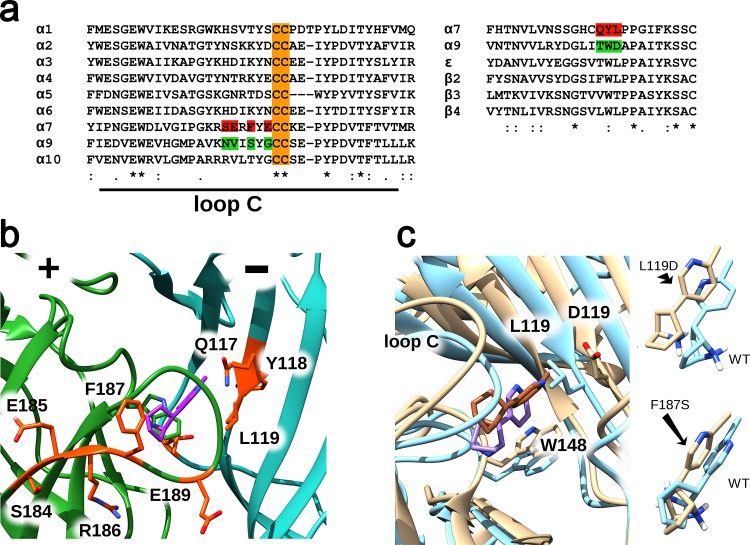
Structural basis for point mutagenesis. (a) Alignments of the loop C region of α-subunits (principal side) and complementary regions of α7, α9, β-, and ɛ-subunits. Mutated residues in the α7-subunit are highlighted in *red*, substituting amino acids from the α9-subunit are highlighted in *green*, conserved cysteines in loop C are highlighted in *orange*. (b) Overall view of the epibatidine (*purple*) binding site located at the subunit interface of the α7 nAChR extracellular part under the loop C. The principal part (“+”) of the α7 nAChR binding site is highlighted in *dark green*, the complementary one (“-“) in *cyan*; the residues picked for mutagenesis are shown in *orange*. Pictures were rendered in UCSF Chimera using PDB 3SQ6. (c) Molecular dynamics study of possible effects of the chosen point mutations on the α7 extracellular domain-epibatidine complex structure (PDB 3SQ6). Among all tried mutations L119D showed the most drastic changes in epibatidine positioning. Other mutations had tiny effects on epibatidine positioning with the exception of F187S that favored an unusual turn of the epibatidine amine group.

To check the role of chosen non-conserved amino acid residues (Fig 1A, *green*) in the unique α9 nAChR pharmacology, several α7/α9 nAChR mutants were prepared, where single amino-acid residues of the α7 nAChR ligand-binding site (Fig 1A, *red*) were replaced by the homologous ones from α9 nAChR (Fig 1A, *green*). Target mutations (Fig 1A) were chosen on the basis of the crystal complex between the α7 nAChR extracellular domain (a chimera with acetylcholine-binding protein) and agonist epibatidine [24] (Fig 1B), and its molecular dynamics study (Fig 1C). Amino acids in positions 117, 118, 119, 184, 185, 187, and 189 (Fig 1B, *orange*) were suggested to be good candidates for substitution as they are found in close proximity to bound epibatidine (Fig 1B, *purple*). Molecular dynamics allowed us to estimate how these suggested point mutations could affect the structure and stability of the α7 extracellular domain-epibatidine complex, resulting in two favorites, L119D and F187S (Fig 1C). The L119D mutation led to the most drastic changes in the epibatidine position, and F187S favored an unusual turn of epibatidine's amine group (Fig 1C). Thus, some residues, such as L119 and F187 might play a crucial role in epibatidine binding to the receptor due to direct influence on the complex structure, contributing to pharmacological differences between α7 and α9 nAChRs. Other residues are unlikely to change binding properties of epibatidine, and may or may not relate to functional differences between these receptors.

### Expression of α7 nAChR mutants

**Fig 2:**
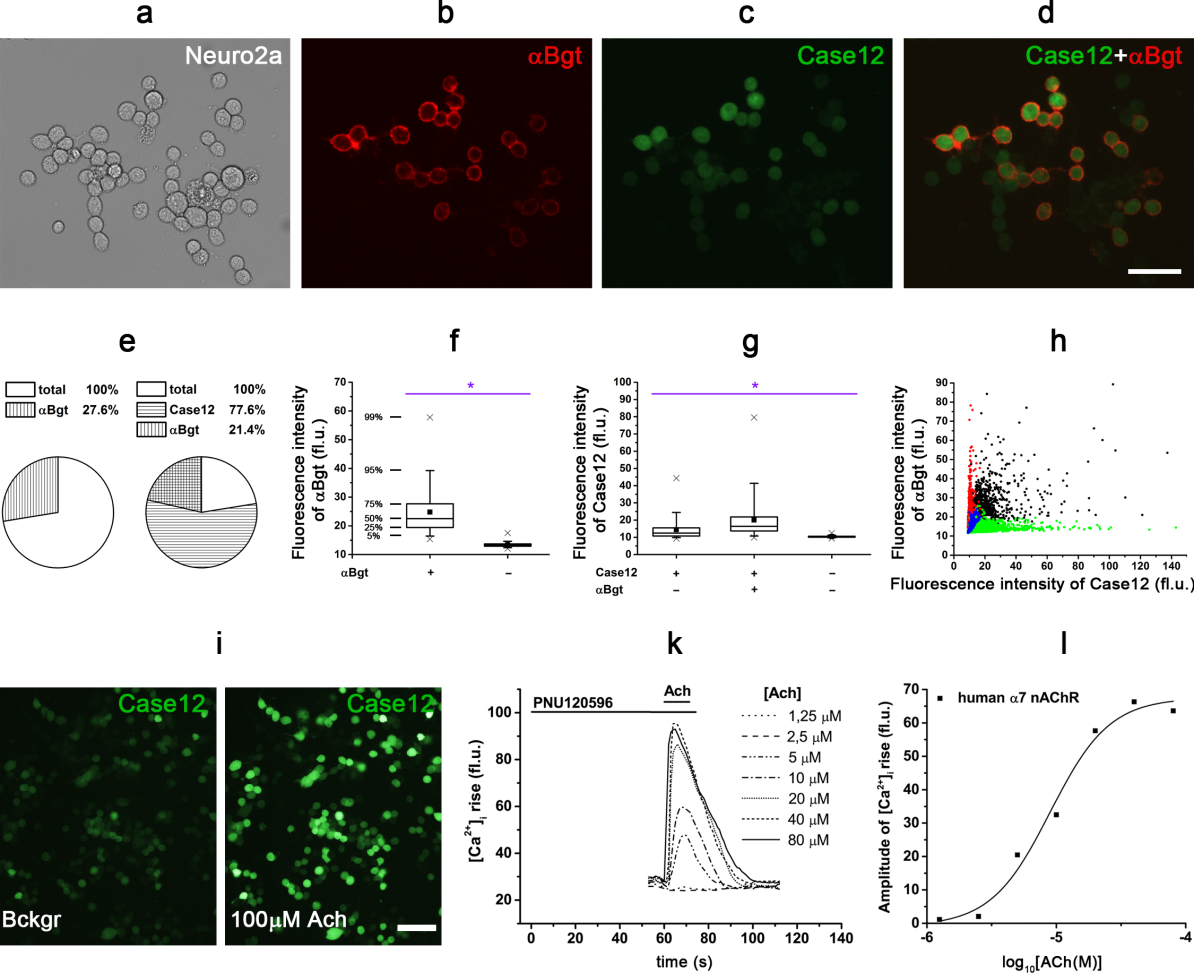
Functional expression of human α7 nAChR in the presence of the chaperone NACHO and the genetically-encoded fluorescent calcium ion sensor Case12 in mouse neuroblastoma Neuro2a cells. (a, b) Cytochemical detection of α7 nAChR with 50 nM Alexa Fluor 555-α-bungarotoxin (αBgt, *red*) and (c, d) its co-expression with Case12 (*green*) in Neuro2a cells. (e) Pie charts represent the percentage of transfected Neuro2a cells labeled with Alexa Fluor 555-α-bungarotoxin (αBgt) in the absence (n = 3, 2521 (total) and 696 (αBgt) cells) or in the presence of Case12 (n = 3, 3141 (total), 2464 (Case12), and 745 (αBgt) cells). (f) Box chart of fluorescence intensity of Alexa Fluor 555-α-bungarotoxin (αBgt) cellular labeling in comparison to background level (n = 3, 1215 and 7641 cells, respectively, Student’s *t*-test, *p<0.05). (g) Box chart of fluorescence intensity of Case12 in the total cell population and in αBgt-positive cells, and intensity of background cellular fluorescence (n = 3, 8315, 1217, and 7643 cells, respectively, one-way ANOVA, *p<0.05). (h) Correlation between fluorescence intensities of Alexa Fluor 555-α-bungarotoxin (αBgt) and Case12 in co-labeled Neuro2a cells (*black points*, weak correlation, *r* = 0.4, Pearson correlation test, *p* = 2.2e^-16^, n = 3, 1217 cells). *Red* and *green* points represent fluorescence intensities of αBgt and Case12 in mono-labeled cell populations, respectively (n = 3, 1209 and 8315 cells, respectively). *Blue* points demonstrate background level of fluorescence intensities (n = 3, 7643 cells). Representative (i) microscopic images (scale bar, 100 μm) and (k) a plot of the intracellular calcium response ([Ca^2+^]_i_ rise) of Neuro2a cell expressing human α7 nAChR, the chaperone NACHO, and the fluorescent calcium ion sensor Case12 to different concentrations of acetylcholine, and (l) corresponding dose-response curve of [Ca^2+^]_i_ rise amplitude. (i) 73.5±1.0% (n = 3, mean±SEM, 1191 cells in total and 880 ACh-responding cells) of Case12-positive cells responded to 100 μM acetylcholine. Neuro2a cells were preincubated with 10 μM PNU120596, a positive allosteric modulator of α7 nAChR, before acetylcholine application. fl.u.–fluorescence units.

To achieve reliable expression of α7 nAChR and its mutants, we performed a transient co-transfection of mouse neuroblastoma cells (Neuro2a) with the plasmids coding for α7 nAChR and one of the chaperones Ric-3 or NACHO. Although α7 nAChR interacts with a number of cellular proteins at different stages of its biosynthesis, folding, assembly, and trafficking, the chaperones Ric-3 and NACHO are of unique importance, constituting an essential requirement for α7 receptor functional expression in mammalian cells [38, 42–45]. The presence of a chaperone significantly increased both human and rat α7 nAChR expression levels and allowed to visualize cell surface-located α7 nAChRs by fluorescent α-bungarotoxin labeling (Fig 2A, 2B and 2D) in 27.6 ± 0.4% cells (mean±SEM, Fig 2E). Intensity of α-bungarotoxin staining differed among cells, but for 95% of the cells it significantly exceeded the background level (Fig 2B, 2D and 2F). In the absence of a chaperone, α-bungarotoxin binding was extremely low, almost undetectable. The specificity of the α-bungarotoxin labeling protocol was knockout-proved earlier [46] and confirmed by the lack of labeling in the presence of 100-fold molar excess of α-cobratoxin, another snake long-chain α-neurotoxin [22].

The expression of several α7 nAChR mutants (Q117T, Y118W, L119D, S184N, E185V) was at a level comparable to that of wild-type (WT) α7 nAChR, according to fluorescent Bgt labeling. The F187S mutant expression was significantly attenuated (about 5% of cells), while with the E189G mutant there was no binding of fluorescent α-bungarotoxin observed. Thus, most mutants were successfully expressed in Neuro2a cells and were labeled with the fluorescent α-bungarotoxin.

### Calcium imaging and electrophysiology of single Neuro2a cells

Pharmacological properties of α7 nAChR WT and mutants were studied by calcium imaging. For this purpose, we performed a transient co-transfection of Neuro2a cells with α7 nAChR (WT or mutant), a chaperone (Ric-3 or NACHO), and the commercially-available fluorescent single-wave calcium ion sensor Case12 genes. Case12-encoding plasmid addition did not decrease the expression capacity of α7 nAChR (Fig 2C, 2D and 2E). Brightness of Case12 fluorescence in α-bungarotoxin-positive cells was not only higher than the background level, but significantly exceeded its average intensity in the total Neuro2a cell population (Fig 2D and 2G). However, we observed only weak correlation between fluorescence intensities of Alexa Fluor 555-α-bungarotoxin and Case12 (*r* = 0.4, Pearson correlation test, *p* = 2.2e^-16^), which might be explained by a strong dependence of Case12 fluorescence on both its expression efficiency and the intracellular calcium ion concentration.

**Fig 3:**
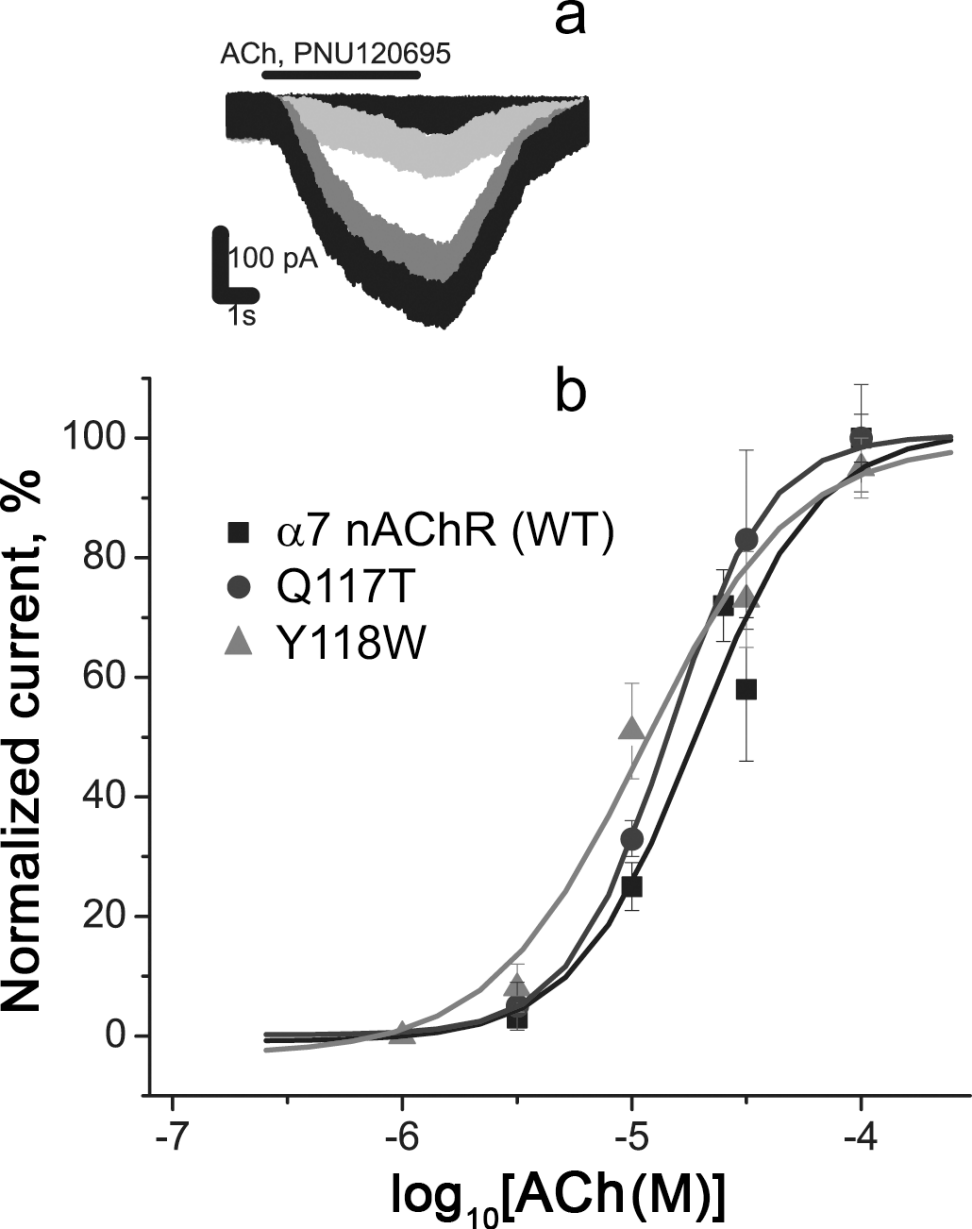
Electrophysiological whole-cell patch clamp recordings of acetylcholine action on α7 nAChR (WT), α7 nAChR [Q117T], and α7 nAChR [Y118W] expressed in Neuro2a cells. (a) Representative currents through α7 nAChR (WT) under application of 3, 10, 30, and 100 μM acetylcholine and (b) dose-response curves. All ligand solutions contained the positive allosteric modulator 10 μM PNU120596, provoking increased and prolonged responses of α7 nAChR to acetylcholine. Each curve was plotted on the basis of averaged data from 6–7 cells (mean ± SD).

In further functional calcium imaging tests, most of the transfected Neuro2a cells responded to specific agonists (acetylcholine and epibatidine), indicating functional expression of α7 nAChRs (WT or mutants) in even higher number of cells (73.5±1.0%, mean±SEM, Fig 2I). This number well-corresponded to the total percentage of Case12-positive cells (77.6±1.5%, mean±SEM, Fig 2E). The observed cell responses originated from the receptor activation, since 2 μM α-cobratoxin, a specific inhibitor of α7 nAChR, blocked them. Using this calcium imaging technique, single-cell responses to application of different concentrations of agonists were recorded (Fig 2I and 2K). Dose-response curves were constructed based on calcium response amplitudes for individual cells (Fig 2L), and the EC_50s_ of acetylcholine were calculated for human WT, Q117T, and Y118W α7 nAChR (Table 2). The obtained calcium imaging results did not reveal any significant differences in the affinity to agonists for WT, Q117T, and Y118W α7 nAChRs (Table 2). These data were in agreement with whole-cell patch clamp electrophysiological recordings performed for the Neuro2a cells (Table 2, Fig 3). In our study, for both calcium imaging and whole-cell patch clamp recordings carried out in Neuro2a cells, we pre-applied a saturating concentration (10 μM) of PNU120596, a type II PAM, before addition of agonists to transfected cells (Figs 2K and 3A). This saturating concentration was chosen on the basis of previously published data [33, 37]. This modulator binds to a specific intrasubunit transmembrane allosteric site of α7 nAChR [47–50], so that the mutations inserted in the orthosteric ligand-binding site of α7 nAChR should not affect binding of PNU120596 to the receptor.

**Table 2:** Agonist affinity to human WT α7 nAChR and mutants Q117T and Y118W measured by calcium imaging and electrophysiology of single cells.

Receptor	Calcium imaging	Electrophysiology
	Acetylcholine,	Acetylcholine,
	EC_50_ (mean ± SEM), μM	EC_50_ (mean ± SD), μM
human α7 nAChR	8.17 ± 0.56	19 ± 4
human α7 nAChR [Q117T]	10.19 ± 0.72	14 ± 1
human α7 nAChR [Y118W]	5.96 ± 0.34	11 ± 3

All ligand solution contained 10 μM PNU120596. EC_50_ of acetylcholine and epibatidine was averaged for 76–199 individual Neuro2a cells in 4–7 independent calcium imaging experiments (mean± SEM), and for 6–7 cells in 3 independent electrophysiological experiments (mean± SD).

The remaining α7 nAChR mutants were tested in pilot calcium imaging experiments using a single high concentration of acetylcholine (100 μM). The cell responses mediated by L119D, S184N, E185V, and F187S mutants were registered, but for E189G there was no functional activity in accordance with the previously observed lack of cytochemical labeling with fluorescent α-bungarotoxin. This mutant was excluded from further investigations and instead we prepared the analogous mutant E189A of the chimeric α7/GlyR, which was successfully expressed in *Xenopus oocytes* and had a slightly higher (about 2-fold) affinity to the agonists acetylcholine and nicotine in electrophysiological tests in comparison with WT α7/GlyR (Table 3).

**Table 3:** Agonist affinity to WT and mutant α7 nAChRs measured by calcium imaging of cell populations, and electrophysiology with *Xenopus oocytes*.

**Receptor**	**Calcium imaging**	
	**Acetylcholine,**	**Epibatidine,**
	Mean EC_50_ (95% CI), μM	Mean EC_50_ (95% CI), nM
human α7 nAChR	8.69 (4.79–15.8)	15.42 (10.54–22.56)
human α7 nAChR [Q117T]	9.95 (5.13–19.28)	19.86 (10.95–36.03)
human α7 nAChR [Y118W]	4.69 (2.53–8.71)	8.63 (2.9–25.7)
human α7 nAChR [S184N]	3.36 (2.23–5.08)	5.86 (3.45–10.04)
human α7 nAChR [E185V]	3.18 (2.09–4.83)	12.17 (4.96–29.88)
**human α7 nAChR [F187S]**	**40.85 (15.04–110.95)**	**111.03 (57.78–213.38)**
rat α7 nAChR	6.54 (3.32–12.89)	19.64 (10.26–36.62)
**rat α7 nAChR [L119D]**	**43.36 (24.75–67.25)**	**617.89 (389.06–981.3)**
human α7 nAChR	6.12 (5.64–6.64)*	
**rat α7 nAChR [L119D]**	82.4 (74.46–91.09)*	
	**Electrophysiology**	
	**Acetylcholine,**	**Epibatidine,**
	Mean EC_50_ (95% CI), μM	Mean EC_50_ (95% CI), μM
rat α7 nAChR	192.7 (149.2–248.8)	1.56 (1.38–1.78)
**rat α7 nAChR [L119D]**	**2125.9** (1489.9–3033.3)	**31.79 (24.35–41.5)**
	**Acetylcholine,**	**Nicotine,**
	Mean EC_50_ (95% CI), μM	Mean EC_50_ (95% CI), μM
α7/GlyR	247.8 (158.1–388.6)	34.3 (20.4–57.7)
α7/GlyR [E189A]	123.3 (86.7–175.4)	19.7 (18.2–21.4)

In calcium imaging studies all ligand solutions contained 10 μM PNU120596.

* The calcium ion indicator Fluo-4 (Thermo Fisher Scientific, USA) was applied instead of the genetically encoded sensor Case12. 95% CI– 95% confidence interval.

**Fig 4:**
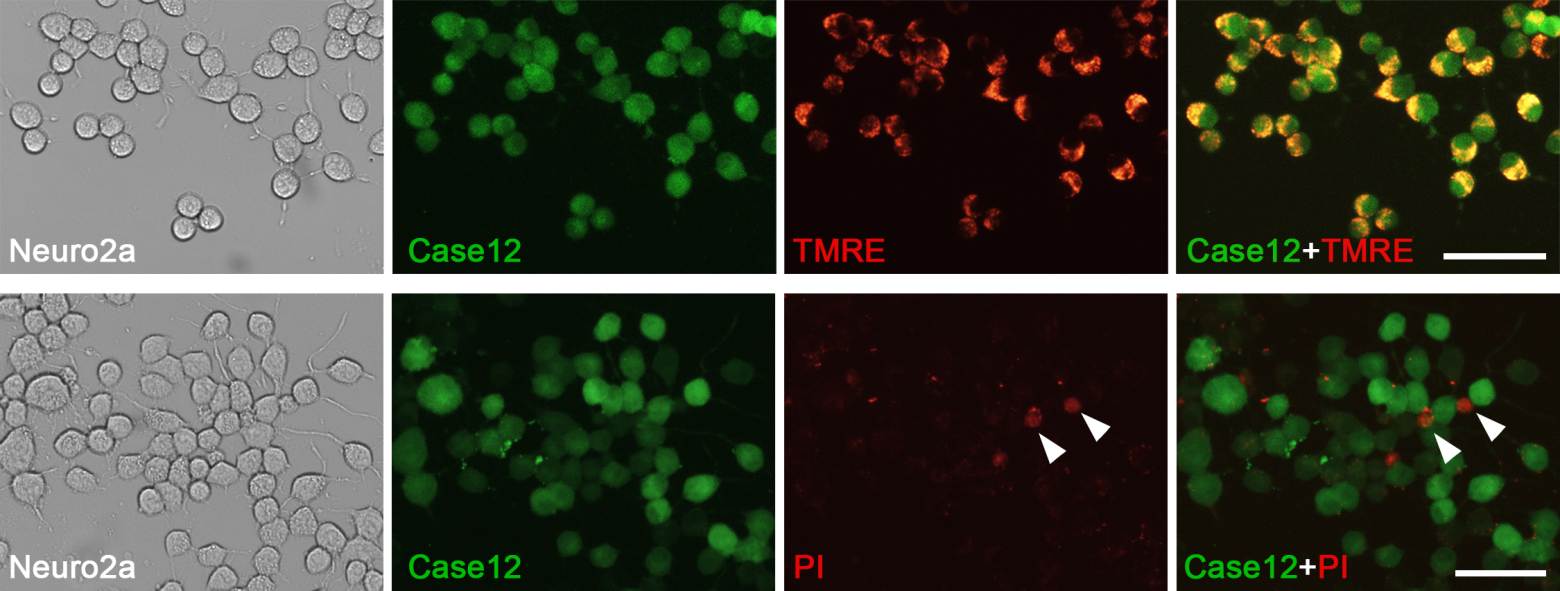
Expression of the fluorescent calcium ion sensor Case12 in Neuro2a cells correlates with cell viability markers. Cytochemistry of Neuro2a cells transfected with plasmids coding human α7 nAChR, the chaperone NACHO, and the calcium sensor Case12 revealed that 93.5±0.7% (mean±SEM) of Case12-positive cells (*green*) were labeled with a cell viability marker 20 nM tetramethylrhodamine ethyl ester (TMRE, top panel, *red*, n = 3,1240 cells). Case12 fluorescence was absent in non-viable Neuro2a cells stained with the DNA-binding reagent propidium iodide (50 ng/ml, bottom panel, *red*, *arrow heads*, n = 3, 1561 cells). Scale bars, 60 μm.

The level of cell viability after the transfection procedure was high, since 93.5±0.7% of Case12-positive cells were alive according to staining with the specific marker—tetramethylrhodamine ethyl ester (TMRE, Fig 4, top panel). In contrast, we did not identify any co-localization of Case12 and propidium iodide, the marker of dead cells (Fig 4, bottom panel). Thus, Case12 was convenient as the indicator of cell viability.

To compare pharmacological properties of all functionally active α7 nAChR mutants in detail we applied a high-throughput screening (HTS) method based on the presented calcium imaging protocol by expanding it from single cells to entire cell populations.

### Calcium imaging of Neuro2a cell populations

Calcium responses of transfected Neuro2a cell populations grown on black 96-well plates were detected with a fluorometric imaging plate reader. Pre-application time with 10 μM PNU120596 was increased to 20 min, since longer periods did not result in higher calcium responses.

**Fig 5:**
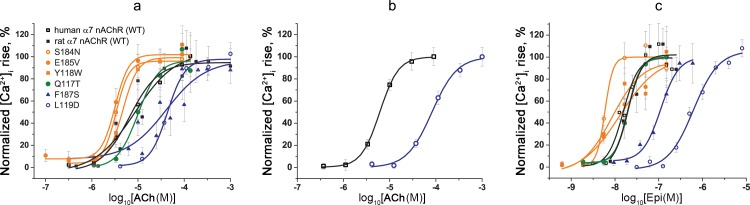
**Dose-response curves of the [Ca^2+^]_i_ rise amplitude in Neuro2a cells expressing WT and mutant α7 nAChRs in response to different concentrations of (a, b) acetylcholine and (c) epibatidine.** The protein calcium sensor Case12 (a, c) and the fluorescent dye Fluo-4 (b) were used to register changes in [Ca^2+^]_i_ in Neuro2a cells. The cells were preincubated with 10 μM PNU120596, a positive allosteric modulator of α7 nAChR, for 20 minutes before agonist application. Each plot point reflects data obtained from 4 independent experiments (mean ± SEM).

Dose-response curves of integrated cellular calcium responses revealed similar affinity of both human and rat α7 nAChR (WT) and Q117T mutant to the tested agonists (Table 3)—acetylcholine (Fig 5A) and epibatidine (Fig 5C). Their mean EC_50s_ for epibatidine were in the range 15.4–19.9 nM, which corresponded well to previously published data for WT α7 nAChR (17–28 nM) [37]. Y118W, S184N, and E185V mutants showed slightly increased affinities for both agonists, although 95% confidence intervals of mean EC_50_ overlapped for WT and those mutant receptors (Table 3, Fig 5A and 5C). In contrast, mutations F187S and L119D caused a significant drop in the affinity to acetylcholine and epibatidine (Table 3, Fig 5A and 5C). While α7 nAChR [F187S] became 5-7-fold less sensitive to both agonists, α7 nAChR [L119D] had approximately 5- and 40-fold lower affinities to acetylcholine and epibatidine, respectively, as compared to the WT receptor (Table 3). Calcium imaging with the conventional fluorescent dye Fluo-4 confirmed pharmacological parameters of the L119D mutant and WT α7 nAChRs (Table 3, Fig 5B). The application of Fluo-4 did not eliminate the necessity for PAM (10 μM PNU120596) addition to ligand solutions to achieve a detectable agonist-induced [Ca^2+^]_i_ rise in Neuro2a cells expressing the receptor of interest.

### Electrophysiological analysis of WT and L119D α7 nAChRs

**Fig 6:**
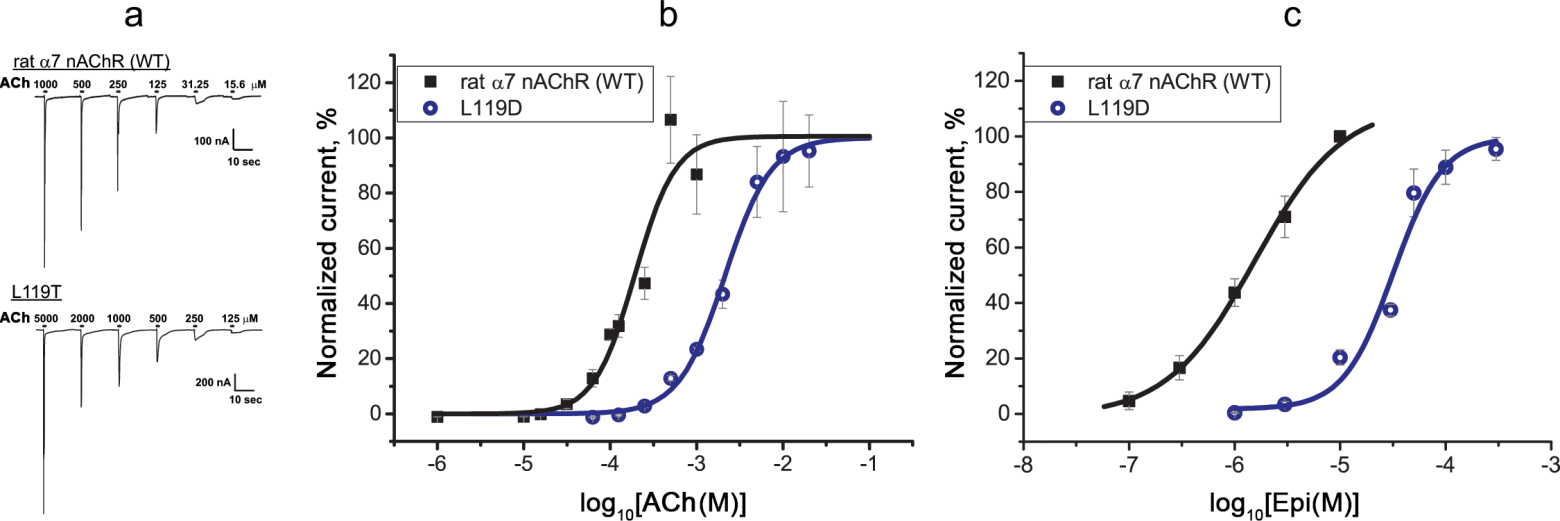
**Electrophysiological recordings of (a, b) acetylcholine- and (c) epibatidine-evoked currents mediated by WT and the L119D mutant α7 nAChRs expressed in *Xenopus* oocytes.** (a) Representative current traces and (b, c) dose-response curves of ion currents. Each plot point reflects data obtained from 5–6 oocytes (mean ± SEM).

To test functional activity of the L119D mutant and WT α7 nAChRs without a positive modulator, we carried out electrophysiology using *Xenopus* oocytes (Fig 6). This analysis confirmed a significantly decreased affinity of α7 nAChR[L119D] to acetylcholine (Fig 6A and 6B) and especially to epibatidine (Fig 6C) compared to WT α7 nAChR (Table 3). Although absolute EC_50s_ of acetylcholine and epibatidine for both receptors obtained by calcium imaging assays in Neuro2a cells and by electrophysiology in *Xenopus* oocytes differed (Table 3), since the action of PAM can change the parameters of ligand-receptor interaction significantly [33], the degree of affinity decrease calculated for the L119D mutant versus WT receptor remained almost identical.

### Calcium imaging analysis of WT and mutant muscle nAChRs

**Fig 7:**
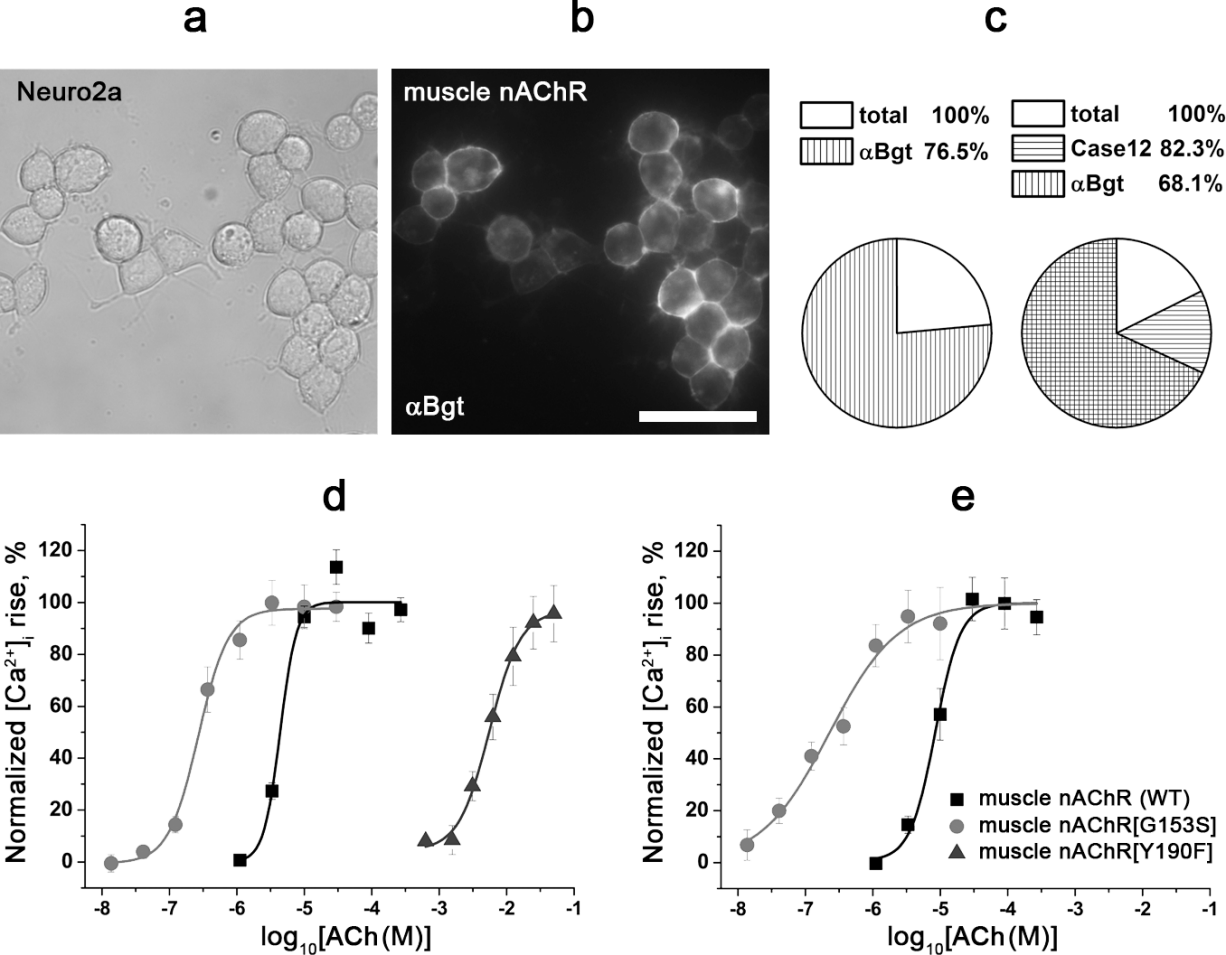
Cytochemistry and calcium imaging of Neuro2a cells expressing WT and G153S, Y190F mutant muscle nAChRs. (a, b) Cytochemical labeling of WT muscle nAChR with Alexa Fluor 555-α-bungarotoxin (50 nM, αBgt) in Neuro2a cells. (a) Bright field image, (b) fluorescent image. Scale bar, 50 μm. (c) Pie charts represent percentage of transfected Neuro2a cells labeled with Alexa Fluor 555-α-bungarotoxin (αBgt) in the absence (n = 3, 413 total cells and 311 cells labeled with αBgt) or in the presence of Case12 (n = 3, 1233 total cells, 1005 cells expressing Case12, and 834 cells labeled with αBgt), respectively. (d, e) Dose-response curves of the [Ca^2+^]_i_ rise amplitude in cells expressing WT and G153S, Y190F mutant muscle nAChRs in response to different concentrations of acetylcholine. The protein calcium sensor Case12 (d) and the fluorescent dye Fluo-4 (e) were used to register changes in [Ca^2+^]_i_. Each plot point reflects data obtained from 4 independent experiments (mean ± SEM).

Further, we studied the applicability of the tested calcium imaging assay to WT and mutant muscle nAChRs. In contrast to α7 nAChR, here was no need in co-expression of a chaperone or application of a modulator; the results were directly compared with previous electrophysiological measurements. Judging from labeling with the fluorescent α-bungarotoxin, most of the transfected Neuro2a cells expressed corresponding receptors (WT and mutant muscle nAChRs with substitutions G153S or Y190F in the α subunit): 76.5±2.1% and 68.1±4.2% of cells were labeled with Alexa Fluor 555-α-bungarotoxin in the absence and in the presence of Case12, respectively (mean±SEM, Fig 7A–7C). As expected, the well-known mutants G153S and Y190F of the muscle nAChR manifest significantly increasing and decreasing affinities to acetylcholine versus the WT receptor, respectively (Table 4, Fig 7D). Performing calcium imaging with Fluo-4 we observed the same pharmacological properties for the receptors tested (Table 4, Fig 7E). The obtained constants (Table 4) were similar to previously published EC_50s_ of acetylcholine for WT and G153S, respectively (2 μM and 51 nM, respectively) [51]. Thus, calcium imaging with the genetically encoded fluorescent calcium sensor Case12 is applicable for a fast analysis of both α7/α9 and muscle nAChR mutants.

**Table 4:** Agonist affinity to WT and mutant muscle nAChRs measured by calcium imaging of cell populations with calcium ion indicators Case12 and Fluo-4.

Receptor	Calcium imaging	
	Acetylcholine,	
	Mean EC_50_ (95% confidence interval)	
	Case12	Fluo-4
muscle nAChR	4.39 (3.18–6.06) μM	8.47 (6.24–11.51) μM
muscle nAChR [G153S]	0.27 (0.19–0.39) μM	0.23 (0.08–0.62) μM
muscle nAChR [Y190F]	5.58 (3.8–8.2) mM	

## Discussion

In the present study, we assessed effects of single amino acid substitutions in the orthosteric ligand-binding site of α7 nAChR by the homologous ones of α9 nAChR on receptor activity. A series of α7/α9 nAChR mutants were produced and their pharmacology tested using the developed calcium imaging technique. This method is based on the transient co-transfection of mouse neuroblastoma cells (Neuro2a) with genetic material of the mutants, in combination with a chaperone Ric-3 or NACHO and a genetically-encoded single-wavelength calcium sensor Case12. To decrease the receptor desensitization rate, PNU120596, an allosteric modulator of α7 nAChR, was co-applied with all ligands. This combination was probed for both microscopic calcium imaging analysis and for a high-throughput screening (HTS) with a fluorometric imaging plate reader (FLIPR). Calcium imaging analysis with the sensor Case12 could also serve for testing other experimental objects such as the muscle nAChR and its mutants.

Nicotinic receptor subunits α7 and α9 are closely related phylogenetically, and some authors considered that they might be homologues of ancestral nAChRs [52]. Our phylogenetic analysis showed that these subunits, along with α10, form a separate clade of the vertebrate nAChR tree. Uniquely, subunits of this clade (α7 and α9) can form both homo- and heteropentamers in contrast to other heteropentameric members of nAChR family [53]. It seems rather unusual that classical nicotinic agonists nicotine and epibatidine act in an opposite manner on such structurally similar α7 and α9 nAChRs. In the present work, a crystallized complex of a chimeric α7 nAChR extracellular domain and epibatidine [24] served as a basis for the molecular dynamics study, which gave us a series of positions expected to be involved in ligand binding. There were two hits, the L119D and F187S mutations, which in the present work by calcium imaging and electrophysiological experiments were confirmed to exert considerable effects on the agonist affinity.

Here, calcium imaging analysis with a genetically-encoded sensor Case12 was preferred for HTS of mutant pharmacology, since the addition of its genetic material along with the receptor and chaperone genes did not complicate the cell transfection procedure. On the contrary, the presence of Case12 allowed us to estimate cell transfection efficiency and cell viability directly, and to perform calcium imaging without such additional procedures as loading cells with a fluorescent calcium indicator, washing of dye excess, etc. Besides, the results obtained with Case12 were confirmed in control experiments with the conventional calcium sensor Fluo-4. Although such commercially available fluorescent dyes are widely used for HTS of ligand-gated ion channel affinity [39, 54, 55], their drawbacks are high cost, variations in dye administration, dye shelf life, cell perturbations during loading, and necessity of cytotoxic inhibitors (e.g. probenecid) of cell transporters to prevent the efflux of intracellular dyes. Another promising tool for HTS are sensor cell lines stably expressing a calcium-permeable LGIC and a genetically encoded FRET (Förster resonance energy transfer)-based calcium sensor [37]. Although this assay lacks some disadvantages of fluorescent dyes, the production of such stable cell lines is challenging and the fluorescence detection system needs to be adapted for the FRET technique. On the contrary, the response of Case12 fluorescence to [Ca^2+^]_i_ oscillations can be measured with a standard filter combination for GFP detection.

The validity of the proposed calcium imaging method was verified by determining affinities to acetylcholine and epibatidine for human and rat α7 nAChRs, as well for a number of α7/α9 mutants and, in addition, by comparison of the results obtained with those from electrophysiology or from a conventional calcium imaging assay. α9 nAChRs are closely related to α7 nAChRs, as α9, α10, and α7 receptors form a separate clade of the vertebrate nAChR phylogenetic tree. For both human and rat α7 nAChRs, the affinities to agonists measured in the presence of PNU120596 were similar and corresponded to those reported in the literature [33, 37]. Calcium imaging of single cells and cell populations, as well as electrophysiological recordings did not reveal any significant difference in the affinity of Q117T and Y118W mutants to agonists in comparison with WT α7 nAChR. In contrast, the position 117 of α7 nAChR was shown to be important for binding of such antagonist as α-conotoxin ImI, but not α-conotoxin PnIB or α-bungarotoxin [56, 57]. A slight tendency of higher sensitivity to agonists was observed for α7 nAChR[S184N], α7 nAChR[E185V], and α7/GlyR[E189A]. These results are in agreement with the previous study, where the different amino acid substitutions at the same positions, namely α7 nAChR[E181S] and α7 nAChR[E185Q] (E185S and E189Q according to our numbering, respectively) significantly increased the receptor affinity to acetylcholine [24]. The strongest changes were observed in the present work for 187 and 119 mutants in comparison with WT receptor: α7 nAChR[F187S] appeared to be 5-7-fold less sensitive to both agonists, and α7 nAChR[L119D] showed approximately 5- and 40-fold lower affinity to acetylcholine and epibatidine, respectively. The F187 residue forms contacts with P194 in the C-loop of the receptor, according to the published X-ray structure of the complex of the α7 nAChR extracellular domain (a chimera with acetylcholine-binding protein) and agonist epibatidine [24] and our molecular dynamics study. Aromatic-proline interactions play important role in protein stability [58] and might be implicated in the C-loop movement required for α7 nAChR gating. Interestingly, α7 is the only human nAChR α-subunit that has such an aromatic-proline pair (see Fig 1). The L119 residue in the complementary face of ligand-binding pocket appears to be important for agonist binding not only to α7 nAChR, but also to β2* and probably even to β4* receptors [59]. The observed more prominent decline in the ability of epibatidine versus acetylcholine to evoke an α7 nAChR[L119D] response might indicate a possible involvement of this amino acid residue in the pharmacological differences between α7 and α9 nAChRs. These results correspond to a recent study showing a crucial role of the D121 residue in the α9 nAChR subunit (in contrast to the homologous L119 in the α7 subunit) for high-affinity binding of its specific antagonist α-conotoxin RgIA [60].

A general applicability of the proposed calcium imaging protocol was demonstrated with the muscle nAChR mutants. Mutation G153S in the α subunit is connected with slow-channel myasthenic syndrome, which is characterized by increased acetylcholine sensitivity [51]. In contrast, mutation Y190F is known to reduce dramatically the affinity to acetylcholine of muscle nAChR [61]. In our study, calcium imaging analyses with the Case12 protein sensor and the Fluo-4 conventional dye confirmed the corresponding literature data for both G153S and Y190F mutants. Pharmacological parameters obtained with both indicators were very similar. Thus, the calcium imaging protocol based on co-expression of a receptor of interest and the protein calcium sensor Case12 can be applied to evaluate functional responses of different nAChRs.

In summary, the proposed calcium imaging technique allowed us to test the functional activity of a number of α7 nAChR mutants with single amino acid substitutions made in the orthosteric ligand-binding site. The strongest changes (decline) in affinity to agonists were demonstrated for mutations at positions 187 and 119. A considerably larger decrease in the affinity of the L119D mutant for epibatidine than for acetylcholine indicates that this position might be among those implicated in the differences in pharmacological properties between α7 and α9 nAChRs.
